# A Novel Entropy-Based Approach for Thermal Image Segmentation Using Multilevel Thresholding

**DOI:** 10.3390/e27050526

**Published:** 2025-05-14

**Authors:** Thaweesak Trongtirakul, Karen Panetta, Artyom M. Grigoryan, Sos S. Agaian

**Affiliations:** 1 Department of Electrical Engineering, Faculty of Industrial Education, Rajamangala University of Technology Phra Nakhon, Bangkok 10300, Thailand; 2 School of Engineering, Tufts University, Medford, MA 02155, USA; karen@ece.tufts.edu; 3 Department of Electrical and Computer Engineering, The University of Texas at San Antonio, San Antonio, TX 78249, USA; 4 College of Staten Island and the Graduate Center, City University of New York (CUNY), Staten Island, NY 10314, USA; sos.agaian@csi.cuny.edu

**Keywords:** entropy, thermal images, segmentation

## Abstract

Image segmentation is a fundamental challenge in computer vision, transforming complex image representations into meaningful, analyzable components. While entropy-based multilevel thresholding techniques, including Otsu, Shannon, fuzzy, Tsallis, Renyi, and Kapur approaches, have shown potential in image segmentation, they encounter significant limitations when processing thermal images, such as poor spatial resolution, low contrast, lack of color and texture information, and susceptibility to noise and background clutter. This paper introduces a novel adaptive unsupervised entropy algorithm (A-Entropy) to enhance multilevel thresholding for thermal image segmentation. Our key contributions include (i) an image-dependent thermal enhancement technique specifically designed for thermal images to improve visibility and contrast in regions of interest, (ii) a so-called A-Entropy concept for unsupervised thermal image thresholding, and (iii) a comprehensive evaluation using the Benchmarking IR Dataset for Surveillance with Aerial Intelligence (BIRDSAI). Experimental results demonstrate the superiority of our proposal compared to other state-of-the-art methods on the BIRDSAI dataset, which comprises both real and synthetic thermal images with substantial variations in scale, contrast, background clutter, and noise. Comparative analysis indicates improved segmentation accuracy and robustness compared to traditional entropy-based methods. The framework’s versatility suggests promising applications in brain tumor detection, optical character recognition, thermal energy leakage detection, and face recognition.

## 1. Introduction

Entropy, a concept introduced by Clausius in 1865 to quantify unusable energy in thermodynamic systems, has since become a cornerstone of modern science. Boltzmann, Gibbs, and others later provided an atomic interpretation of entropy within statistical mechanics and gas dynamics, establishing its foundational role in describing non-equilibrium processes through the second law of thermodynamics and the principle of maximum entropy production. In the mid-20th century, Claude E. Shannon redefined entropy as a measure of uncertainty or randomness in datasets, laying the groundwork for its application in information theory. Today, entropy and entropic forces are integral to innovative approaches in artificial intelligence and the study of collective behavior, underscoring their significance across diverse scientific disciplines.

Entropy has become a powerful tool for quantifying an image’s complexity in image processing. It is widely employed in compression, segmentation, quality assessment, and feature extraction tasks. A high entropy value generally indicates a complex image featuring a broad range of pixel values, while a low entropy value implies a more straightforward, more uniform image [1]. For example, entropy analysis can assess an image’s complexity to assist in identifying the best compression method without substantial information loss [2]. By quantifying the randomness or uncertainty in pixel values, entropy provides insights into the quantity of redundant or compressible information in an image, facilitating efficient data storage and transmission [3]. Likewise, entropy plays a role in image quality assessment and feature extraction. High entropy values often indicate more detailed and complex images, which are typically richer in information [4,5]. This property makes entropy a valuable metric for assessing image quality, as higher entropy generally correlates with greater detail and visual complexity. In feature extraction, entropy helps identify information-rich areas within an image. Regions with high entropy are often prioritized for further analysis because they likely contain significant features or patterns of interest [6,7]. This capability is beneficial in applications like object detection, segmentation, and pattern recognition, where it is crucial to distinguish meaningful regions from the background [8].

This paper focuses on applying entropy-based techniques to image segmentation, particularly in identifying illegal activities from images captured by thermal infrared (TIR) cameras in challenging environments [9,10,11]. Monitoring protected areas to reduce illegal activities, like poaching and wildlife trafficking, poses a significant and complex challenge [12]. These activities threaten biodiversity and disrupt the ecological balance, undermining global conservation efforts. Effective management and surveillance require advanced technologies, such as remote sensing, drones, and artificial intelligence (AI), to enhance the detection, prevention, and response to these threats [13,14]. Addressing these issues is crucial for ensuring the long-term conservation of biodiversity and the sustainability of ecosystems.

Recent advancements in aerial imaging technologies have resulted in widespread use of satellite and Unmanned Aerial Vehicle (UAV)-based data for various applications, such as surveillance and monitoring. Nevertheless, imagery captured in the visible spectrum often faces limitations in low-light environments or adverse weather conditions [15,16]. The growing interest in using sensors in the near-infrared (NIR) and thermal infrared (TIR) spectrums, driven by significant cost reductions, has enabled improved detection and tracking capabilities in more challenging environments. This advancement marks a significant step forward in enhancing surveillance and management of protected areas [17,18].

Detecting illegal activities from TIR images in challenging environments requires an efficient digital signal processing framework, especially using segmentation techniques. These methods aid in differentiating and identifying suspicious objects against the terrain in the captured imagery [19]. This segmentation is essential for identifying illegal activities, such as human movement in forested areas or the use of hunting tools [20]. Using digital signal processing for segmentation makes it possible to enhance the detection and tracking of these activities, reducing the image analysis’s complexity. Furthermore, this approach boosts the operational efficiency of law enforcement by speeding up the response time to potential threats or illegal actions [21,22].

Segmentation primarily focuses on isolating regions of interest, such as heat signatures from humans or animals, which may indicate illegal activities like poaching or logging. The goal is to differentiate these heat sources from the background, which typically represents the natural environment or terrain. One commonly used method for image segmentation is thresholding, where pixel values are categorized into distinct segments based on a predefined threshold. However, identifying the optimal threshold can be challenging, especially without prior knowledge of the image’s content. Various extensions of entropy, including Boltzmann–Gibbs, Tsallis, Renyi, Kapur, and Masi entropies, have been developed and employed for image segmentation [1,23,24,25,26,27,28,29,30,31,32]. These entropy measures offer unique advantages and disadvantages [32,33,34,35].

In 1985, Kapur introduced entropy [29] as a popular method for image segmentation. However, it has several limitations: (i) Sensitivity to noise, particularly in low-contrast or noisy images [36]. TIR (thermal infrared) images, which often contain thermal noise or artifacts, can produce inaccurate entropy calculations, resulting in suboptimal thresholding and poor segmentation performance. (ii) The assumption of a bimodal distribution of pixel intensities, meaning it is most effective when the image can be divided into two distinct regions (e.g., foreground and background). However, the method may struggle to generate accurate thresholds in complex images with multi-modal intensity distributions, such as those featuring varying heat sources or cluttered backgrounds [37]. (iii) Computational complexity. Calculating the entropy for each potential threshold value requires evaluating the distribution of pixel intensities across multiple segments, which can be computationally demanding, especially for large images or real-time applications. This may limit the scalability and efficiency of Kapur entropy in large-scale surveillance systems or scenarios that need rapid processing [38]. (iv) Limited contextual awareness. The method considers only the statistical distribution of pixel intensities without incorporating spatial information or contextual relationships between pixels, potentially missing critical structural details in the image. (v) Parameter sensitivity. The effectiveness of entropy-based methods can be highly dependent on parameter selection, requiring careful tuning for optimal performance across different imaging conditions [39].

Moreover, Kapur’s entropy significantly relies on the assumption that the background and foreground can be separated solely based on intensity differences. In some cases, the thermal signatures of objects may overlap with the background, particularly in dynamic environments or under varying thermal conditions, which can diminish the effectiveness of segmentation. A key limitation of Kapur entropy for segmentation is that it often necessitates preprocessing steps, such as image enhancement, to improve its effectiveness. Since Kapur entropy is grounded in intensity thresholding, it assumes that the regions of interest (like the heat signatures of humans or animals) are distinguishable from the background due to their thermal characteristics. However, TIR images may frequently suffer from low contrast or poor visibility due to environmental noise, fluctuating thermal conditions, or similar temperature values in the foreground and background.

To address these challenges, this paper proposes a novel entropy-based multilevel thresholding approach for improved thermal image segmentation. The proposed method is evaluated on the BIRDSAI datasets [40], benchmarking the automatic detection and tracking of humans and animals in both real and synthetic videos. The key contributions of this work are as follows:

- Image enhancement techniques specifically designed for TIR images improve visibility and contrast in regions of interest. These methods emphasize the thermal signatures of objects, such as humans or animals, making them more distinguishable from the background. This step is essential for enhancing the performance of subsequent segmentation processes.
- An innovative entropy-based segmentation technique tailored for TIR images is presented. The proposed method employs advanced entropy measures to determine the optimal multilevel threshold, enabling more precise separation of foreground and background regions, even in challenging, low-contrast TIR images.

Through these contributions, we aim to enhance the detection and monitoring of illegal activities in natural environments using TIR imaging, thereby supporting global conservation efforts. The remainder of this paper is structured as follows: Section 2 provides a comprehensive overview of entropy-based segmentation techniques. Section 3 presents the proposed methodology, including image enhancement techniques for TIR images and the innovative entropy-based segmentation approach. Section 4 showcases the results of computer simulations, followed by a discussion of the findings. Finally, Section 5 concludes the paper with a summary of the contributions and suggests potential directions for future research.

## 2. Background

Image segmentation is a fundamental task in computer vision and image processing, enabling the extraction of meaningful regions of interest (ROIs) from complex visual data. Among the various segmentation techniques, entropy-based methods have gained prominence for their ability to quantify uncertainty and randomness in pixel intensity distributions, making them especially effective for thresholding applications. This section reviews key entropy formulations and their roles in segmentation, focusing on thermal infrared (TIR) imaging, where isolating heat signatures from noisy backgrounds remains a significant challenge. In this paper, we define the ROIs within a thermal image $\left(X_{a,b}\right)$ as the set of pixels that meet a predefined entropy-based thresholding criterion (T):

(1) $$\Omega =\left\{\left(a,b\right)|X_{a,b}\geq T_{\Phi }\right\},$$

where $T_{\Phi }$ is the optimal threshold, as defined in Section 2.2.

### 2.1. Entropy in Image Segmentation

Entropy, which comes from information theory, measures the unpredictability or dispersion of data. In imaging, it quantifies the variability in pixel intensities, with higher entropy values indicating greater randomness. Several formulations of entropy have been adapted for segmentation:

(2) $$H(x)=-\sum_{i}p\left(x_{i}\right)\log\left(p\left(x_{i}\right)\right),$$

where $p\left(x_{i}\right)$ is the probability of intensity $x_{i}$. It underpins thresholding techniques by maximizing the total entropy of segmented regions (e.g., foreground vs. background). While effective for basic segmentation, Shannon entropy assumes separable intensity distributions and struggles with multi-modal or noisy data.

***Tsallis Entropy*:** A generalization of Boltzmann–Gibbs entropy [41], Tsallis entropy [42] introduces a non-extensive parameter, q. This parameter enables tuning for sensitivity to multi-modal distributions, making it suitable for complex images. However, its performance hinges on the careful selection of q.

(3) $$H(x)=\frac{1}{q-1}\left(1-\sum_{i}p^{q}\left(x_{i}\right)\right),$$

***Renyi Entropy*:** it extends Shannon entropy with a parameter α α to emphasize sparsity or concentration [43]:

(4) $$H(x)=\frac{1}{1-\alpha }\left(\log\left(\sum_{i}p^{\alpha }\left(x_{i}\right)\right)\right),$$

While adaptable to subtle intensity variations, its computational complexity limits scalability.

***Kapur Entropy*:** it optimizes thresholding for bimodal histograms by splitting intensities into the foreground $\left(H_{0}\right)$ and background $\left(H_{1}\right)$ [4]:

(5) $$H_{0}\left(x\right)=-\sum_{i=0}^{T}\frac{p\left(x_{i}\right)}{P_{0}}\log\left(\frac{p\left(x_{i}\right)}{P_{0}}\right);\;\;\;H_{1}\left(x\right)=-\sum_{i=T+1}^{L-1}\frac{p\left(x_{i}\right)}{P_{1}}\log\left(\frac{p\left(x_{i}\right)}{P_{1}}\right),$$

where $P_{0}$ and $P_{1}$ are cumulative probabilities. Despite its simplicity, Kapur entropy is sensitive to noise and fails for multi-modal or overlapping distributions.

***Masi Entropy*:** it introduces a flexibility parameter, r, to handle complex distributions [44]:

(6) $$H_{0}\left(x\right)=\frac{1}{1-r}\log\left[1-\left(1-r\right)\sum_{i=0}^{T}\frac{p\left(x_{i}\right)}{P_{0}}\log\left(\frac{p\left(x_{i}\right)}{P_{0}}\right)\right]\mathrm{and}\;H_{1}\left(x\right)=\frac{1}{1-r}\log\left[1-\left(1-r\right)\sum_{i=T}^{L-1}\frac{p\left(x_{i}\right)}{P_{1}}\log\left(\frac{p\left(x_{i}\right)}{P_{1}}\right)\right]$$

Though promising for diverse intensity profiles, its real-time applicability remains underexplored.

### 2.2. Thresholding Techniques and Challenges

Multilevel thresholding techniques have been widely studied using entropy-based methods, such as Shannon, Kapur, and Tsallis entropy. These approaches leverage statistical information to determine optimal threshold values for segmentation. Meta-heuristic optimization techniques like Genetic Algorithms (GAs) and Particle Swarm Optimization (PSO) strive to find optimal or near-optimal solutions to complex optimization problems where traditional methods may fall short as shown in Table 1. For threshold selection specifically, these techniques have the following objectives:

- Identify optimal threshold values that maximize segmentation accuracy;
- Decrease computational complexity compared to exhaustive search methods;
- Prevent getting trapped in local optima in a complex fitness landscape;
- Address multi-dimensional optimization problems involving multiple thresholds.

Application to Threshold Selection: For threshold selection in image segmentation or signal processing, these meta-heuristics can automatically determine optimal thresholds without an exhaustive search; adapt to different image characteristics and noise conditions; optimize multiple criteria simultaneously (e.g., between-class variance and entropy); and scale to multilevel thresholding problems more efficiently than traditional methods. Both the GA and PSO have proven effective for threshold selection, with the choice between them typically depending on the specific application constraints, the computational resources available, and the complexity of the fitness landscape. However, recent advancements have leveraged meta-heuristic optimization techniques such as the Genetic Algorithm (GA) [45] and Particle Swarm Optimization (PSO) [46] to enhance threshold selection efficiency [47]. These methods iteratively refine threshold positions using evolutionary strategies, often resulting in improved segmentation accuracy. However, their computational cost is significantly higher than that of entropy-based methods.

#### 2.2.1. Bilevel vs. Multilevel Thresholding

Bilevel thresholding partitions an image into two classes (e.g., object and background) by optimizing entropy at a single threshold. For multilevel thresholding, the intensity histogram is divided into n intervals using thresholds $T_{1},T_{2},\ldots ,T_{n}$, maximizing the joint entropy:

(7) $$T_{\Phi }=\underset{0<T_{1}<\ldots <T_{n}<L-1}{\mathrm{argmax}}\left(H_{0}+H_{1}+\ldots +H_{n}\right),$$

while multilevel approaches better handle complex images, the computational complexity escalates exponentially, posing a significant optimization challenge.

#### 2.2.2. Limitations in Thermal Infrared (TIR) Imaging

Thermal infrared imaging is crucial for applications such as surveillance, medical diagnostics, and environmental monitoring, where isolating heat signatures from cluttered backgrounds is essential. However, TIR images inherently exhibit low contrast, thermal noise, and overlapping intensity profiles between foreground objects (e.g., humans and machinery) and backgrounds. These challenges complicate segmentation, as traditional intensity-based methods often struggle to distinguish regions of interest (ROIs) under such conditions. Kapur entropy, a widely used method for thresholding, maximizes the entropy of segmented regions to identify an optimal threshold. Applying entropy to bilevel thresholding involves selecting a threshold that maximizes the entropy of segmented regions. While effective for simple cases, bilevel thresholding is limited when handling images with multiple regions of interest.

To address the limitations of bilevel thresholding in complex TIR images, multilevel thresholding partitions the intensity of the histogram into multiple intervals, each representing a distinct region (e.g., background, human, and machinery). Advanced entropy measures, including Tsallis and Renyi, are employed to optimize thresholds. For Kapur entropy, the multilevel extension is defined as

(8) $$H_{total}=\sum_{k=0}^{n}H_{k}\;\;\;;\;\;\;H_{k}=-\sum_{i=T_{k}}^{T_{k+1}}\log\left(\frac{p\left(x_{i}\right)}{P_{k}}\right),$$

where $P_{k}$ denotes the cumulative probability of the k-th class.

While this approach enhances the segmentation of multi-modal TIR data, the inherent limitations of Kapur entropy, including noise sensitivity and computational cost, persist, particularly as the number of thresholds increases. Additionally, Kapur’s entropy relies heavily on the assumption that the background and foreground can be separated solely based on intensity differences. In specific scenarios, the thermal signatures of objects may overlap with the background, particularly in dynamic environments or under varying thermal conditions, which can diminish the effectiveness of segmentation. A notable limitation of Kapur entropy for segmentation is its frequent requirement for preprocessing steps, such as image enhancement, to boost its effectiveness. Since Kapur entropy depends on intensity-based thresholding, it assumes that regions of interest (such as the heat signatures of humans or animals) are distinguishable from the background based on their thermal characteristics. However, TIR images may encounter low contrast or poor visibility in many situations due to environmental noise, varying thermal conditions, or comparable temperature values in the foreground and background.

To address the issues mentioned, image enhancement techniques are often necessary to highlight the region of interest and improve the contrast between foreground objects and the background. Without such preprocessing, the Kapur entropy method may struggle to accurately determine an optimal threshold, leading to suboptimal segmentation outcomes. Therefore, image enhancement techniques like contrast adjustment, noise reduction, or filtering are crucial to ensuring that the segmentation process based on Kapur entropy produces reliable and meaningful results.

### 2.3. A-Entropy

As mentioned above, entropy is widely used to measure uncertainty or information content in image processing. Shannon entropy is a fundamental concept in information theory that quantifies uncertainty or randomness in a system, providing a measure of a source’s information content. Shannon entropy has extensive data compression, communication systems, and image processing applications. Shannon entropy $\left(H_{s}\right)$ for a discrete random variable i with possible outcomes $\left\{i_{1},i_{2},..,i_{n}\right\}$ and corresponding probabilities $p_{i}$ is given by

(9) $$H_{s}=-\sum_{i=1}^{n}p_{i}\log\left(p_{i}\right),$$

where $p_{i}$ denotes the global probability density function of the occurrence of the outcome i.

**Figure 1:**
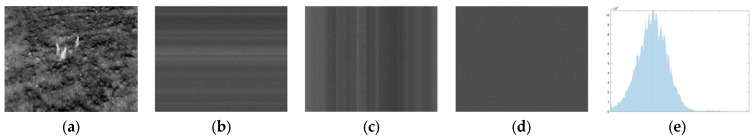
Comparison of pixel shuffling in grayscale image with entropy and standard deviation values: (**a**) original image; (**b**) image with pixels shuffled row-wise; (**c**) image with pixels shuffled column-wise; (**d**) fully shuffled image (rows and columns); (**e**) image histogram.

Meanwhile, Shannon entropy highlights a significant limitation in spatial awareness. Current entropy models, such as Shannon, Tsallis, and Renyi entropy, are calculated only from the probability distribution function (PDF) of pixel intensities, overlooking the spatial arrangement of those pixels. Consequently, when the pixels in an image are randomly shuffled, the PDF remains unchanged, resulting in identical entropy values for both the original and shuffled images. This limitation persists even though the two images are visually distinct, as demonstrated in Figure 1.

To illustrate the limitation of global statistical image quality assessments (IQAs), Table 2 presents the inadequacy of global metrics, where the standard deviation (σ), Shannon entropy $\left(H_{s}\right)$, Tsallis entropy $\left(H_{t}\right)$, and Renyi entropy $\left(H_{r}\right)$ are compared before and after pixel shuffling. Despite the loss of structural coherence, these metrics remain unchanged, as they are influenced solely by the statistical distribution of pixel intensities.

#### 2.3.1. Block-Based Probability Density Functions (BPDFs)

The new model is specifically designed to overcome the spatial insensitivity of traditional entropy measures by integrating local spatial information within block-based regions. This approach provides a comprehensive representation of image content, allowing better differentiation between visually distinct images with the same global intensity histograms. The formulation of the proposed A-Entropy initiated by Agaian is

(10) $$H=\sum_{i=1}^{n}\left[{\left(\frac{{\left[p_{i}\right]}_{x,y}^{\Omega }}{{\left[\omega \right]}_{x,y}^{\Omega }}+\varepsilon \right)}^{\gamma }\tan^{\gamma }\left(\log\left(\frac{{\left[p_{i}\right]}_{x,y}^{\Omega }}{{\left[\omega \right]}_{x,y}^{\Omega }}+\varepsilon \right)\right)\right],$$

where ${\left[p_{i}\right]}_{x,y}^{\Omega }$ denotes the probability density of pixel intensities within a block Ω, ${\left[\omega \right]}_{x,y}^{\Omega }$ refers to a weighting factor to normalize probabilities within each block, γ represents an adjustable parameter that controls sensitivity to probability variations, and ε is a small constant to avoid undefined behavior during computation.

Local PDFs extend the traditional concept of global statistical analysis by dividing the image into localized regions and analyzing the statistical characteristics within each region. By leveraging localized intensity distributions, entropy-based analyses can capture the spread or concentration of intensity values in specific areas, thus addressing the limitations of global metrics. Table 3 presents a comparative analysis of entropy-based metrics using local information.

#### 2.3.2. Monotonic Properties

**Figure 2:**
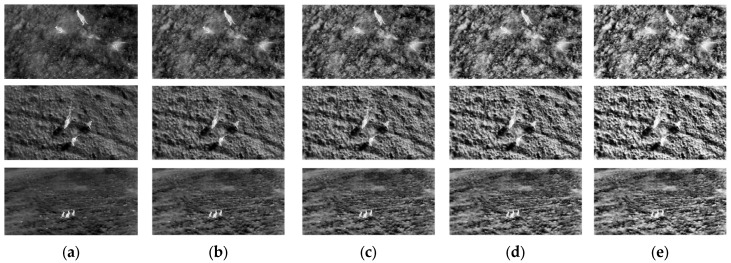
Comparison of thermal image enhancement with different Degrees of Enhancement (DoEs): (**a**) original image; (**b**) 25% DoE; (**c**) 50% DoE; (**d**) 75% DoE; (**e**) 100% DoE.

**Figure 3:**
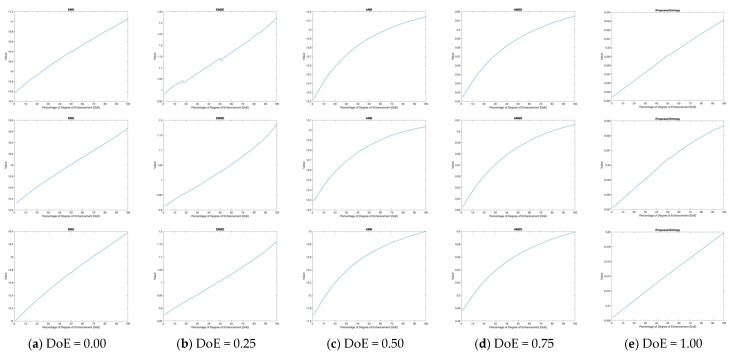
Monotonic increase in kernel-based metric values with higher Degrees of Enhancement (DoEs) depicted in Figure 2: (**a**) original image; (**b**) 25% DoE; (**c**) 50% DoE; (**d**) 75% DoE; (**e**) 100% DoE.

The incorporation of local PDFs demonstrates an improvement in detecting structural changes, making them a more accurate and reliable approach to IQA. In addition, when applied to enhanced images, the metrics measure increases in contrast by analyzing the spread and concentration of intensity values within localized regions. Higher Degree-of-Enhancement (DoE) values correspond to greater contrast improvements, as reflected in entropy-based metrics, as shown in Figure 2 and Figure 3, and Table 4.

The results presented in Table 3 demonstrate the performance of various kernel-based metrics, including Enhancement Measure Estimation (EME), Enhancement Measure Estimation by Entropy (EMEE), Average Michelson Contrast Estimation (AME), Average Michelson Contrast Estimation by Entropy (AMEE), and the proposed entropy-based metric, across images with increasing Degrees of Enhancement (DoEs). These metrics collectively capture the effects of enhancement on image quality in terms of contrast.

The values for all metrics show a consistent monotonic increase with higher DoE levels across all images (*Image1*, *Image2*, and *Image3*), as illustrated in the accompanying figure. This trend confirms the effectiveness of the proposed entropy model, where an increased DoE correlates with improved contrast and structural clarity in the images. The proposed metric steadily rises, more accurately representing structural and contrast changes. This is particularly evident in the uniformity of its response across all tested images.

## 3. Proposed Method

This section introduces an effective method of multi-threshold image segmentation based on entropy. The implementation steps are detailed below.

### 3.1. Entropy-Based Image Segmentation with Adaptive Gamma Correction

Utilizing an entropy-based measure to tackle multi-thresholding challenges in image segmentation involves mapping solutions to problems. By leveraging entropies, measurable uncertainty, or information content metrics, segmentation methods aim to partition images into distinct regions by maximizing information gain. This approach has proven effective in scenarios requiring optimal threshold determination, such as separating foreground from background regions or distinguishing objects of interest within complex scenes. Despite its advantages, the entropy-based method faces limitations, including sensitivity to noise, high computational demands, and reliance on global thresholding strategies. These challenges drive ongoing research aimed at refining entropy-based segmentation techniques. To address these issues, the proposed concept employs the principles of entropy-based image segmentation. The method begins with adaptive gamma correction applied to the input image to enhance contrast and adjust for variations in illumination. This preprocessing step ensures that the image’s dynamic range is optimized for segmentation tasks, making it more suitable for entropy-based analysis.

The motivation behind the proposed method is to integrate entropy-based image segmentation techniques with local probability density functions (PDFs) in both the logarithmic and trigonometric domains. By transforming the image data into these domains, the algorithm can more effectively capture both the global and local characteristics of the image, as shown in Algorithm 1.
**Algorithm 1:** Entropy-based multilevel thresholds.**Input:**   Input thermal image, I, of size M×N. $\;\mathrm{Number}\;\mathrm{of}\;\mathrm{thresholds},\;T_{n}$. **Output:** $\mathrm{Optimal}\;\mathrm{thresholds},\;T_{\Phi }$.$\mathrm{Normalize}\;\mathrm{intensity}\;\mathrm{value}:\;I_{norm}\leftarrow \frac{I}{\max\left\{I\right\}}$ $\mathrm{Compute}\;\mathrm{histogram}:\;h_{i}\leftarrow \sum_{x=1}^{M}\sum_{y=1}^{N}\delta \left(I_{norm}\left(x,y\right)-i\right)\;;\forall i\in \left[0,L-1\right]$, where L              denotes the number of intensity levels and δ refers to the Dirac             delta function. $\mathrm{Compute}\;\mathrm{PDF}:\;p_{i}\leftarrow \frac{h_{i}}{\sum_{i=0}^{L-1}h_{i}}$ $\mathrm{Compute}\;\mathrm{the}\;\mathrm{cumulative}\;\mathrm{weight}:\;\omega_{s}\leftarrow \sum_{i\in s}p_{i}$$,\;\mathrm{where}\;\omega_{s}$ represents the cumulative                 weight of segment s. $\mathrm{Define}\;\mathrm{the}\;\mathrm{entropy}\;\mathrm{function}:\;E_{s}\leftarrow -\sum_{i\in s}\left(\frac{p_{i}}{\omega_{s}}+\varepsilon \right)\cdot \tan\left(\log\left(\frac{p_{i}}{\omega_{s}}+\varepsilon \right)\right)$, where ε is a constant. $\mathrm{Initialization}:\;E_{max}\leftarrow -\infty$$\;\mathrm{and}\;T_{\Phi }\leftarrow \left\{0,0,...,0\right\}$$\;\mathrm{of}\;\mathrm{size}\;T_{n}$. For a=1 **Do**     For b=a+1 **Do**     **For** … **Do** $\mathrm{Partition}\;\mathrm{the}\;\mathrm{intensity}\;\mathrm{range}\;\mathrm{into}\;T_{n}$ segments: ${\;S}_{k}\leftarrow \left\{\begin{array}{c} \left\{i|t_{k-1}\leq i\leq t_{k}\right\}\;,\;\;k\leftarrow 1,2,\ldots ,T_{a}\\ \left\{i|t_{k-1}\leq i\leq t_{k}\right\}\;,\;\;k\leftarrow T_{a+1},\ldots ,T_{b}\\ \ldots \\ \left\{i|t_{k-1}\leq i\leq t_{k}\right\}\;,\;\;k\leftarrow T_{n-1},\ldots ,T_{n} \end{array}\right.$$\;\mathrm{where}\;T_{0}\leftarrow 0$. Compute the total entropy for the current threshold: ${\;E}_{T}\leftarrow \sum_{k=1}^{T_{a}}E\left(S_{k}\right)+\ldots +\sum_{k=T_{n}}^{L-1}E\left(S_{k}\right)$ $\;\mathrm{If}\;\;E_{T}>E_{max}$ $\;E_{max}\leftarrow E_{T}$ $\;T_{\Phi }\leftarrow T_{a},T_{b},\ldots ,T_{n}$          **End**       **End**   **End** **End**

Algorithm 1 outlines the detailed computational process of the proposed multilevel entropy-based thresholding method, providing a structured framework for guiding the segmentation procedure. By integrating this approach with the iterative multilevel thresholding technique, the segmentation process is further enhanced through the iterative refinement of threshold values. This refinement, driven by entropy optimization, ensures more accurate segmentation of regions within the image, particularly for complex images with varying intensity distributions, as detailed in Algorithm 2.

To maintain computational stability, the initial entropy value is set to −∞, as shown in Algorithm 1. Additionally, to prevent division errors when $p_{i}=0$, the calculation is skipped by setting ε to 1, as defined in the entropy function in Algorithm 1.


**Algorithm 2:** Iterative multilevel thresholding image segmentation.**Input:**   Input thermal image, I, of size M×N. $\;\mathrm{Number}\;\mathrm{of}\;\mathrm{thresholds},\;T_{n}$. **Output:** Segmented image, B.$T_{\Phi }\leftarrow$$\;\mathrm{Call}\;\mathrm{Algorithm}\;I\;(I,T_{n}\leftarrow 1)$. $\mathrm{Calculate}\;\mathrm{the}\;\mathrm{mean}\;\mathrm{intensity}:\;\mu \leftarrow \frac{\sum_{i\in \left\{I>T_{\Phi }\right\}}I_{i}}{Countof\;\left\{I>T_{\Phi }\right\}}$ Compute γ:$\;\gamma \leftarrow e^{\left(\frac{\mu -\frac{\max\left\{I\right\}}{2}}{\frac{\max\left\{I\right\}}{2}}\right)}$ $\mathrm{Generate}\;a\;\mathrm{mapping}\;\mathrm{function}:\;f\left(i\right)\leftarrow \log\left(\frac{\left(\frac{i}{L-1}\right)}{1-\left(\frac{i}{L-1}\right)}+\gamma \right)$ $\mathrm{Rescale}\;f\left(i\right)$$\;\mathrm{to}\;\left[0,L-1\right]:$$\;f^{\prime }\left(i\right)\leftarrow \left(\frac{f\left(i\right)-\min\left\{f\left(i\right)\right\}}{\max\left\{f\left(i\right)\right\}-\min\left\{f\left(i\right)\right\}}\right)\cdot \rho \cdot \left(L-1\right)$ $\mathrm{Apply}\;f^{\prime }\left(i\right)$$\;\mathrm{to}\;\mathrm{the}\;\mathrm{image}:\;Y\leftarrow f^{\prime }\left(I_{i}\right)\;;\forall i\in \left\{1,2,\ldots ,M\times N\right\}$ $T_{\Phi }\leftarrow$$\;\mathrm{Call}\;\mathrm{Algorithm}\;I\;(Y,T_{n}\leftarrow n)$. Partition the intensity into n+1$\;\mathrm{segments}:\;S_{k}\leftarrow \left\{\begin{array}{c} \left\{p|p\leq t_{1}\right\}\;\;\;\;\;\;\;\;\;\;\;\;\;\;\;,\;\;k\leftarrow 1\\ \left\{p|t_{k-1}<p\leq t_{k}\right\}\;,\;\;k\leftarrow 2,\ldots ,n\\ \left\{p|p>t_{n}\right\}\;\;\;\;\;\;\;\;\;\;\;\;\;\;\;,\;\;k\leftarrow n+1 \end{array}\right.$ $\mathrm{Compute}\;\mathrm{the}\;\mathrm{local}\;\mathrm{mean}\;\mathrm{for}\;\mathrm{each}\;S_{k}$$:\;\mu_{k}\leftarrow \frac{\sum_{p\in S_{k}}p}{\left|S_{k}\right|}$ $\mathrm{Assign}\;\mu_{k}$$\;\mathrm{to}\;\mathrm{all}\;\mathrm{pixels}\;\mathrm{in}\;S_{k}:$$\;G_{p}\leftarrow \mu_{k}$ $T_{\Phi }\leftarrow$$\;\mathrm{Call}\;\mathrm{Algorithm}\;I\;(G,T_{n}\leftarrow 1)$. Binarize G$\;\mathrm{using}\;T_{\Phi }:$$\;B\leftarrow \left\{\begin{array}{c} 0,\;\;G_{p}\leq T_{\Phi }\\ 1,\;\;G_{p}>T_{\Phi } \end{array}\right.$


This algorithm performs multilevel thresholding-based segmentation on thermal images using an iterative approach. The primary objective is to adaptively enhance the foreground, efficiently remove the background, and reduce the computational complexity of threshold determination.

### 3.2. Adaptive Image Enhancement

**Figure 4:**
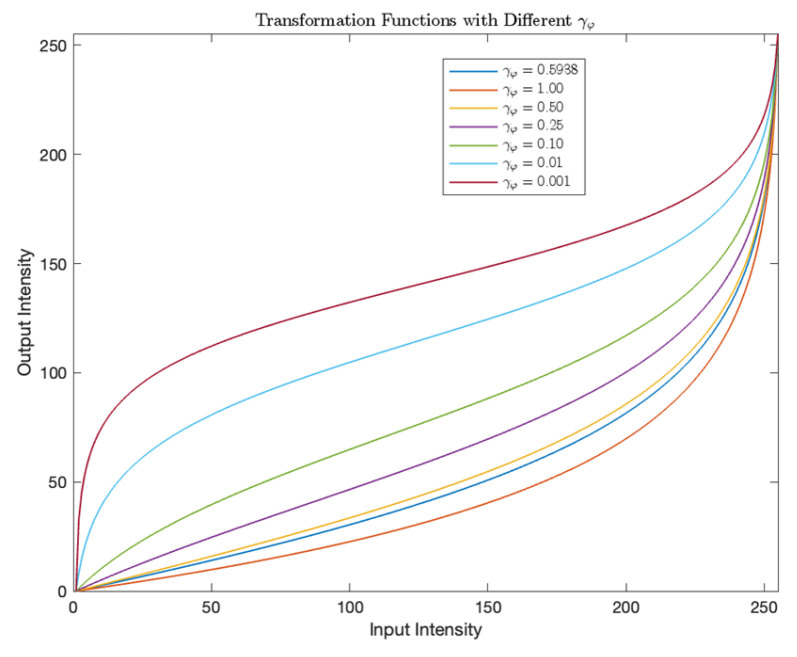
Transformation functions with different $\gamma_{\varphi }$.

The proposed algorithm introduces an adaptive image enhancement framework designed to improve the visibility and contrast of bright regions while minimizing irrelevant background details. Unlike conventional image enhancement techniques that rely on statistical histogram-based values, this method dynamically adjusts the gamma parameter $\left(\gamma_{\varphi }\right)$ according to the global intensity properties of the input image. The gamma value is computed using the global mean intensity $\left(\mu \right)$ and the maximum intensity value $\left(I_{max}\right)$ of the image, as defined by

(11) $$\gamma_{\varphi }=e^{\left(\frac{\mu -I_{max}/2}{I_{max}/2}\right)},$$

here, μ represents the global mean intensity of an input image $\left(I\right)$, enabling the algorithm to adapt to varying lighting and contrast conditions. This adaptive computation ensures that the gamma value aligns with the image’s inherent characteristics, preventing over- or under-enhancement. Following the gamma calculation, a logit-based transformation is applied to amplify foreground homogeneity and suppress background noise. The transformation function is expressed as

(12) $$f\left(i\right)=\log\left(\frac{i/\left(L-1\right)}{1-i/\left(L-1\right)}+\gamma_{\varphi }\right)+\rho ,$$

where i is the pixel intensity, L is the total number of intensity levels (e.g., 256 for an 8-bit image), $\gamma_{\varphi }$ is the adaptive parameter from Equation (11), and ρ is a constant. This function enhances contrast by non-linearly redistributing intensity values (see Figure 4), emphasizing subtle differences in foreground regions while attenuating background variations. The proposed image enhancement technique achieves a balanced improvement tailored to the specific content of the image by integrating global intensity statistics with a logit transformation. This approach optimizes the balance between foreground visibility and background suppression, enabling more accurate segmentation and analysis.

**Figure 5:**
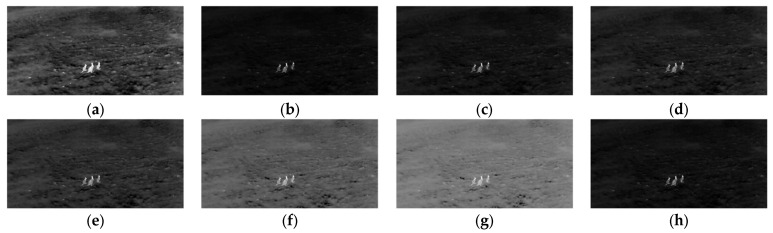
Comparison of thermal image with different γ: (**a**) input image; (**b**) γ=1.00; (**c**) γ=0.50; (**d**) γ=0.25; (**e**) γ=0.10; (**f**) γ=0.01; (**g**) γ=0.001; (**h**) $\gamma =e^{\left(\frac{\mu -\max\left\{I\right\}/2}{\max\left\{I\right\}/2}\right)}$.

The experimental results validate the efficacy of the proposed image enhancement approach in optimizing image segmentation tasks. Figure 5 illustrates how dynamically computed gamma values refine the balance between foreground enhancement and background suppression. When gamma values are set lower than the proposed adaptive parameter, the transformation creates a pronounced separation between the foreground and background. For example, in Figure 5h, the kangaroo (region of interest) is accentuated by brighter intensities, while the background is uniformly darkened, minimizing distractions and improving focus on critical structures. This selective enhancement directly enhances segmentation accuracy by amplifying contrast gradients between the foreground and background, even in low-contrast scenarios.

**Figure 6:**
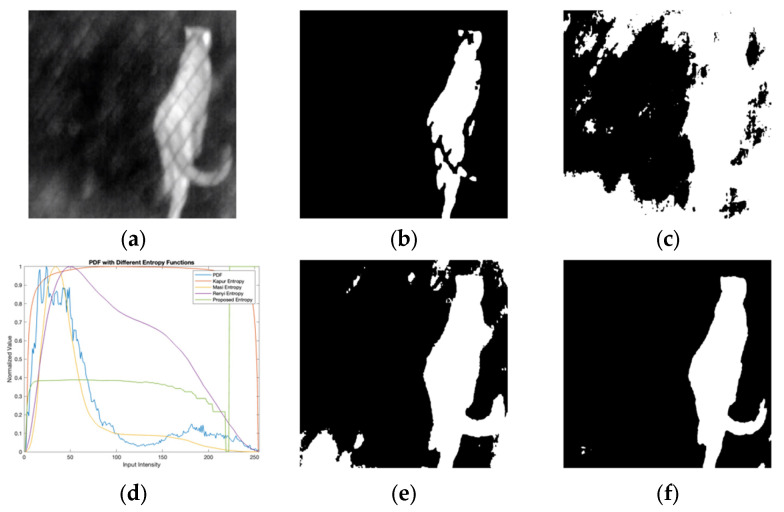
Comparative analysis of segmentation accuracy between the proposed model and existing entropy-based functions: (**a**) input image; (**b**) Kapur segmentation; (**c**) Masi segmentation; (**d**) entropy functions; (**e**) Renyi segmentation; (**f**) proposed segmentation.

Figure 6 compares the proposed method’s segmentation outcomes with traditional entropy-based approaches (Kapur, Masi, and Renyi) on images with complex intensity distributions. Key observations include the following: (i) Kapur’s method is prone to over-segmentation, especially in regions with high intensity variability, resulting in fragmented outputs that lack structural coherence; (ii) Masi’s method struggles with boundary delineation, failing to capture precise object contours; and (iii) Renyi’s method improves object definition compared to Kapur and Masi methods but retains residual noise and compromises structural continuity. In contrast, the proposed method achieves clean, cohesive segmentation by leveraging adaptive image enhancement. It effectively suppresses noise, isolates primary objects, and preserves structural integrity, aligning closely with human perceptual expectations. The proposed method also demonstrates superior adaptability to intensity variations through its smooth and distinct probability density function (PDF). Unlike traditional entropy-based techniques, which exhibit erratic PDFs in heterogeneous regions, the adaptive image enhancement ensures a balanced intensity redistribution. This results in a more robust segmentation framework capable of handling complex lighting and contrast conditions.

**Figure 7:**
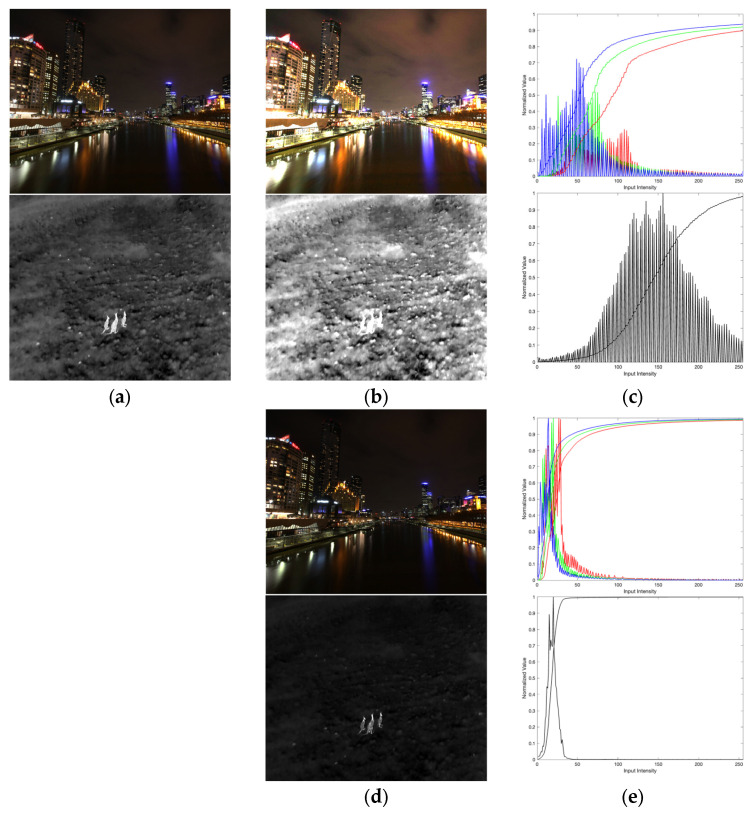
Comparative analysis of enhancement performance with different (**a**) input images; (**b**) enhanced images ρ=μ/10γ; (**c**) histogram of (**b**); (**d**) enhanced images ρ=2; (**e**) histogram of (**d**).

Additionally, the comparative analysis in Figure 7 highlights the critical role of the parameter ρ in balancing contrast enhancement and background suppression. When ρ is adaptively derived from the image’s global mean intensity (μ), as in the case of ρ=μ/10γ, as shown in Figure 7b, the enhancement process prioritizes context-aware adjustments. This adaptive setting produces a histogram, as shown in Figure 7c, with a broader intensity distribution, indicating improved dynamic range and contrast in regions of interest. Such adaptive tuning aligns the enhancement with the image’s inherent intensity characteristics, ensuring that foreground details are accentuated without overamplifying noise.

In contrast, a fixed ρ=2, as shown in Figure 7d, results in a more uniform enhancement effect. The corresponding histogram, as shown in Figure 7e, exhibits a narrower intensity spread, suggesting aggressive background suppression at the cost of reduced contrast in mid-tone regions. While this setting effectively darkens non-critical areas, it risks over-smoothing subtle foreground textures, particularly in scenes with low baseline contrast.

## 4. Computer Simulation Results and Discussion

**Figure 8:**
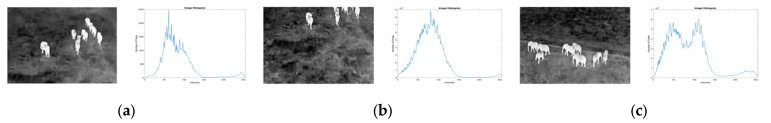
Test images and their histograms for: (**a**) Image4; (**b**) Image5; (**c**) Image6.

We implemented the proposed approach using the computer language MATLAB2024b on a personal computer with 16 GB of memory and a CPI of Apple M2 Pro running macOS Sequoia 15.2 (24C101). To show the advantages of this method, we performed several experiments on the BIRDSAI dataset [40], which is used to detect wildlife from thermal imagery, namely, *Image4*, *Image5*, and *Image6*, as shown in Figure 8. We applied the same preprocessing procedure to all methods before segmentation to ensure a fair comparison. This preprocessing step included adaptive image enhancement to improve segmentation accuracy. We then compared the proposed algorithm with various entropy-based image thresholding methods, including the Shannon [48], Tsallis [42], Renyi [43], Kapur [4], and Masi [44] methods.

### 4.1. Databases

**Figure 9:**
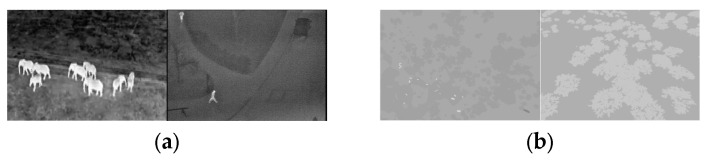
Sample images from the real and synthetic datasets. From left to right: small, medium, and large objects. The two images in (**a**) are real images of animals and humans, respectively, while (**b**) presents synthetic images of animals and humans. The synthetic data comprise a mixture of summer and winter scenes, with winter scenes featuring dark trees against the ground.

The BIRDSAI dataset facilitates research in aerial wildlife monitoring, conservation, and anti-poaching surveillance. It provides a blend of real and synthetic aerial thermal infrared (TIR) images to support domain adaptation and robust algorithm development under challenging visual conditions [33]. Figure 9 shows some sample images from the real and synthetic datasets.

This dataset features aerial TIR images of protected African areas and is designed for object detection, domain adaptation, and tracking of humans and animals. It is the first large-scale dataset from a fixed-wing UAV across multiple African sites, containing 48 real and 124 synthetic videos, totaling 62,000 and 100,000 images, respectively. The data include nine classes: human, elephant, and lion (real and synthetic); giraffe and dog (real); and crocodile, hippo, zebra, and rhino (synthetic). Synthetic data were generated using AirSim with a 3D savanna model and a TIR camera simulation.

Real data were collected using battery-powered fixed-wing UAVs in South Africa, Malawi, and Zimbabwe, with the specific locations withheld for security. Nighttime flights, lasting 1.5–2 h, occurred at altitudes of 60–120 m and speeds of 12–16 m/s, using FLIR Vue Pro 640 and Tamarisk 640 cameras. Temperature conditions varied seasonally, with winter nights ranging from below 0 °C to 4 °C and summer nights from 18 to 20 °C. Challenges included warm ground temperatures, reducing thermal contrast post-sunset, and occasional fog-induced “whiteouts”.

**Figure 10:**
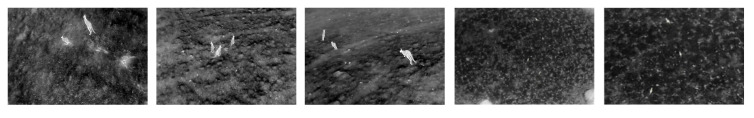
Sample images from the real-world kangaroo (Macropodidae) dataset.

The kangaroo (Macropodidae) dataset [49] provides regularly captured images from thermal imaging surveys. The imagery in this database was collected by the Department of Primary Industry, New South Wales (NSW), and the Department of Primary Industries and Regional Development, Western Australia, as illustrated in Figure 10.

### 4.2. Objective Results

In evaluating the performance of our model, the performance of six methods—Shannon [48], Tsallis [42], Renyi [43], Kapur [4], Masi [44], and the proposed method—was evaluated across six critical metrics: accuracy, Boundary F1 (BF) score, Sørensen–Dice Similarity, Jaccard Similarity, precision, and recall. The evaluation was conducted using three thresholds (*k* = 1, 2, 3) on selected test images (*Image4*, *Image5*, and *Image6*) due to space constraints.

#### 4.2.1. Metric Descriptions

The evaluation of semantic segmentation can be quite complex because it is necessary to measure classification accuracy and localization correctness. The aim is to score the similarity between the predicted (prediction) and annotated segmentation (ground truth). The evaluation metrics used in this paper are summarized in Table 5.

Based on the literature and practical considerations, it is helpful to combine different metrics. Individual metrics such as accuracy, the Dice Similarity Coefficient (DSC), Boundary F1 score (BF), Jaccard Similarity (IoU), precision, and recall each provide unique approaches but also have limitations. For instance, accuracy is simple to compute, but it can be misleading in imbalanced datasets. The DSC and IoU are effective for measuring region overlap but may overlook boundary precision. In contrast, the BF score captures boundary alignment but is less informative about the overall region accuracy. 

Table 6 presents a comparative summary of the strengths and limitations of the key evaluation metrics in image segmentation evaluation [50].

By integrating these advantages and compensating for individual weaknesses, a Combined Score (CS) is introduced. It is calculated by weighting two key metrics: the Dice Similarity Coefficient (DSC) and the Boundary F1 score (BF):

(13) $$CS=\omega_{m}\cdot DSC+\left(1-\omega_{m}\right)BF$$

where $\omega_{m}$ is a constant.

#### 4.2.2. Performance Analysis

Table 7 presents the segmentation accuracy across various entropy-based thresholding methods under different numbers of thresholds. The results indicate that the proposed method achieves the highest accuracy, particularly in the single-threshold case (*k* = 1), with scores of 0.9997, 0.9990, and 0.9978 for Image4, Image5, and Image6, respectively. While Masi is competitive, with scores of 0.9924, 0.9945, and 0.9841, it trails the proposed method. For *k* = 2, the proposed method performs well, with accuracies of 0.9841, 0.9918, and 0.9672, although Kapur and Masi show comparable results, particularly for *Image5* and *Image6*. At *k* = 3, the proposed method maintains high accuracy (0.9751, 0.9877, and 0.9458), although Renyi and Masi slightly outperform it in some cases. Typically, entropy-based segmentation relies on histogram bin separation, and using a single threshold makes it challenging to segment all regions accurately. However, the proposed method demonstrates robust performance even under this constraint.

Table 8 presents the Boundary F1 (BF) scores for various entropy-based thresholding methods across different numbers of thresholds. The results show that Masi delivers the highest boundary precision across all images and threshold settings. Notably, Masi achieves near-perfect scores at *k* = 3, with values of 0.9944 for *Image4*, 0.9964 for *Image5*, and 0.9541 for *Image6*. These results demonstrate Masi’s effectiveness in delineating object boundaries, especially in higher-threshold scenarios.

The proposed method also performs competitively, particularly for a single threshold (*k* = 1), achieving BF scores of 0.9821, 0.8964, and 0.9412 for *Image4*, *Image5*, and *Image6*, respectively. However, its performance slightly declines as the number of thresholds increases, suggesting that it is especially well suited for low-complexity segmentation tasks. For *k* = 2 and *k* = 3, the proposed method trails Masi. This indicates room for improvement in handling more complex multi-threshold segmentation cases. Renyi and Kapur show strong performance in multi-threshold scenarios. For instance, Renyi achieves the highest BF score (0.9986) on *Image5* for *k* = 3. It outperforms the proposed method and closely matches Masi. Kapur also performs well. It reaches scores of up to 0.9877 on *Image5* and *Image6*. In contrast, Shannon and Tsallis exhibit poor boundary performance, especially for multi-threshold settings (*k* = 2 and *k* = 3).

Overall, while Masi leads in boundary accuracy across all settings, the proposed method remains highly competitive for single-threshold segmentation. Its strong performance at *k* = 1 makes it an attractive option for real-time or resource-constrained applications. The low computational overhead further supports its suitability where accurate boundary preservation is essential.

Table 9 presents the Sørensen–Dice Similarity scores for various entropy-based thresholding methods across different numbers of thresholds. The proposed method achieves the highest similarity scores for single-threshold segmentation (*k* = 1) on all three images, with values of 0.9966, 0.9799, and 0.9850 for *Image4*, *Image5*, and *Image6*, respectively. This demonstrates the method’s strong ability to preserve object regions in low-complexity segmentation tasks. However, the performance of the proposed method declines as the number of thresholds increases. At *k* = 2 and *k* = 3, the similarity scores drop significantly, especially for *Image6* (0.7035 and 0.3907, respectively). This suggests that the method is less effective in handling complex segmentation tasks that require multiple thresholds. In contrast, Masi shows strong and consistent performance across all threshold levels. It delivers high Sørensen–Dice scores, particularly at *k* = 3, with values of 0.9517, 0.9408, and 0.9846 across *Image4*, *Image5*, and *Image6*, respectively. Masi’s stability makes it a strong alternative for multi-threshold segmentation scenarios. Renyi and Kapur also perform well in multi-threshold cases, especially at *k* = 3, with Renyi achieving over 0.92 for all images. Kapur shows notable improvement at *k* = 3 on *Image4* and *Image6*. It reaches scores of 0.8551 on *Image4* and 0.8749 on *Image6*. On the other hand, Shannon and Tsallis perform poorly, particularly in multi-threshold settings. Their scores drop sharply with increasing thresholds. This indicates their limited capability in accurately segmenting complex scenes.

In summary, the proposed method excels in single-threshold segmentation. It offers near-perfect similarity to the ground truth with minimal computational demand. However, methods like Masi or Renyi may offer more consistent performance for applications requiring more intricate, multi-threshold segmentation. However, visual comparison offers a similar output. As mentioned above, based on the literature and practical considerations, the above metrics do not always work well for all kinds of image segmentation [50].

Table 10 presents Jaccard Similarity scores for different entropy-based thresholding methods across varying numbers of thresholds. The proposed method achieves the highest scores for a single threshold (*k* = 1) on all images, with values of 0.9933 (*Image4*), 0.9607 (*Image5*), and 0.9704 (*Image6*). This indicates exceptional overlap between the segmented results and the ground truth when using a single threshold, confirming the proposed method’s reliability in low-complexity segmentation tasks. However, as the number of thresholds increases, the performance of the proposed method declines noticeably. For *k* = 2, the scores drop to 0.6761, 0.6515, and 0.5426 for *Image4*, *Image5*, and *Image6*, respectively. At *k* = 3, the scores decrease further, particularly on *Image6*, for which the score reaches only 0.2428. This suggests a significant reduction in segmentation accuracy under complex multi-threshold conditions. In contrast, Masi shows strong and consistent performance across all thresholds, especially at *k* = 3, achieving 0.9079, 0.8882, and 0.9696 for *Image4*, *Image5*, and *Image6*, respectively. This highlights Masi’s robustness in more complex segmentation scenarios. Renyi and Kapur also show improved performance as the number of thresholds increases, particularly on *Image4* and *Image6*. For example, Renyi reaches 0.8785 on *Image5* and 0.8586 on *Image6* at *k* = 3. It outperforms the proposed method in multi-threshold settings. On the other hand, Shannon and Tsallis exhibit weak performance, especially for *k* = 2 and *k* = 3, where scores drop significantly across all images. This indicates their limited suitability for detailed segmentation.

The proposed method excels in single-threshold segmentation with high similarity and low computational cost. This makes it well suited for applications requiring real-time processing and simple segmentation. Alternative methods like Masi or Renyi may offer better performance for complex scenarios involving multiple thresholds.

Table 11 presents precision scores for various entropy-based thresholding methods across different numbers of thresholds (*k* = 1, *k* = 2, and *k* = 3) for three test images (*Image4*, *Image5*, and *Image6*). Across the board, higher precision values reflect more accurate and reliable segmentation performance.

The proposed method exhibits excellent performance, particularly at multi-threshold levels, achieving perfect precision (1.0000) for all images at *k* = 2 and *k* = 3. This indicates that the method is highly effective in minimizing false positives, especially in complex segmentation scenarios. Even at *k* = 1, its precision remains high—0.9933 for *Image4*, 0.9607 for *Image5*, and 0.9704 for *Image6*. This confirms the method’s reliability in simple segmentation tasks and its potential for consistent performance as complexity increases. Masi, Renyi, and Kapur entropies also perform exceptionally well, with Masi and Kapur achieving near-perfect or perfect precision across all thresholds. Renyi entropy slightly underperforms only at *k* = 3 for *Image6* (0.9780) but remains highly competitive. These methods are consistently effective at reducing false positives and maintaining high true-positive detection across various threshold levels. In contrast, Shannon and Tsallis entropies perform well only at *k* = 1, but they suffer from severe drops in precision at higher thresholds. For instance, Shannon’s precision on *Image5* falls to 0.0238 at *k* = 3, and Tsallis exhibits similarly poor performance across images beyond *k* = 1. These results highlight their increased susceptibility to false positive classifications as segmentation complexity grows.

Overall, the proposed method demonstrates outstanding performance in high-precision segmentation, particularly for multi-threshold tasks. It effectively minimizes false positives. Along with Masi, Renyi, and Kapur, it proves suitable for complex segmentation applications. However, given the limitations of precision as a standalone metric, future evaluations should incorporate recall-based measures to ensure a balanced assessment of segmentation quality.

Table 12 presents the recall scores for various entropy-based thresholding methods applied to three test images (*Image4*, *Image5*, and *Image6*) across increasing segmentation complexity (*k* = 1, 2, and 3). Recall quantifies the proportion of correctly identified object pixels (true positives) out of all actual object pixels (true positives + false negatives). A higher recall indicates fewer missed detections, which is especially critical in applications where missing relevant regions is costly, such as medical imaging or defect detection.

The proposed method achieves perfect recall (1.0000) for all three images at *k* = 1. It demonstrates an excellent ability to detect all relevant pixels in low-complexity segmentation. However, its performance degrades as segmentation complexity increases. At *k* = 2, recall drops to 0.6761 (*Image4*), 0.6515 (*Image5*), and 0.5426 (*Image6*) and declines further at *k* = 3, particularly on Image6 (0.2428). This trend suggests that the proposed method minimizes false negatives in simple segmentation tasks. However, its ability to recall all relevant pixels diminishes in multi-threshold conditions. This decline may be attributed to over-segmentation or the application of stricter pixel classification criteria as the number of thresholds increases. In contrast, Shannon and Tsallis entropies exhibit strong recall at higher thresholds. Both methods reach perfect recall (1.0000) for all three images at *k* = 3. Even at *k* = 2, they maintain strong recall scores. This implies that the methods detect nearly all object pixels in complex segmentation scenarios. However, this performance comes despite their previously noted low precision. It indicates a tendency to over-label background pixels as objects. Masi entropy performs well. It balances recall across all thresholds. It achieves scores ranging from 0.8390 to 0.9912. Also, it shows robustness in both complex and straightforward segmentation tasks. This suggests a well-balanced detection strategy that minimizes both false negatives and false positives. Renyi and Kapur entropies, however, yield comparatively lower recall values, particularly at *k* = 1. For instance, Kapur only achieves 0.1637 on Image6 at *k* = 1, while Renyi achieves 0.2428. However, both methods improve recall as *k* increases. They still fall short in overall effectiveness. Neither Renyi nor Kapur matches the performance of Masi or Shannon/Tsallis at higher thresholds. This suggests that their recall improvements are not sufficient to outperform the more robust methods in complex segmentation tasks.

While high recall indicates the effective detection of relevant pixels, it does not penalize false positives. A method with high recall may still suffer from poor segmentation quality if it classifies too many background pixels as objects. Therefore, recall should be interpreted in conjunction with precision or summarized using the F1 score, which provides a harmonic balance of the two metrics.

However, a visual comparison offers a similar output. As mentioned above, based on the literature and practical considerations, the six evaluation metrics do not always perform well across all types of image segmentation tasks. Specifically, in the case of Elephant Segmentation (ES), using accuracy as an evaluation metric is not recommended due to the severe class imbalance between regions of interest (ROIs) and background pixels. In typical ES datasets, ROIs constitute only a small portion of the image, while the background dominates the pixel distribution. Since accuracy includes true negatives—which are abundant in such imbalanced scenarios—it often results in inflated and misleading performance scores. This does not accurately reflect the model’s ability to detect clinically or contextually relevant structures. Therefore, more informative metrics, such as the Dice Similarity Coefficient and Boundary F1 score, are preferred for assessing segmentation quality in this context.

### 4.3. Visual Evaluation

**Figure 11:**
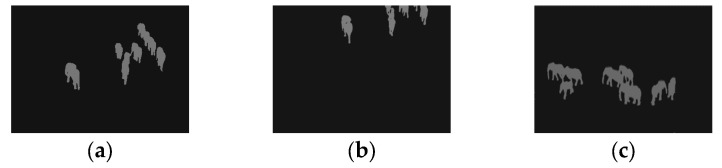
Ground truth: (**a**) *Image4*; (**b**) *Image5*; (**c**) *Image6*.

In this section, the results of the algorithms are visually compared. Due to page length constraints, *Image4*, *Image5*, and *Image6* are selected as representative examples for analysis. The ground truth for these images is presented in Figure 11.

**Figure 12:**
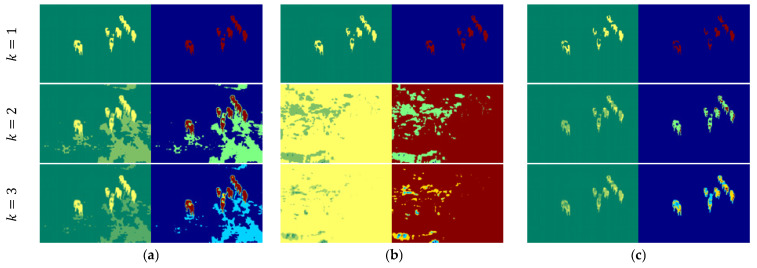

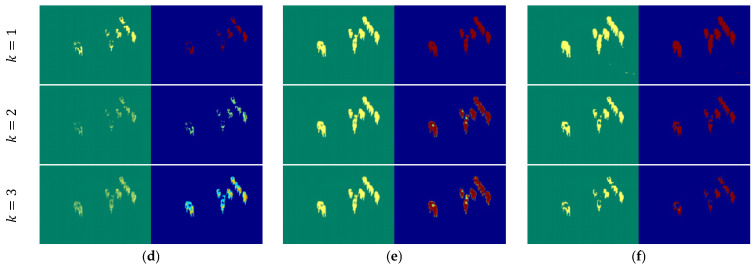
Thresholding results for *Image4*: (**a**) Shannon [48]; (**b**) Tsallis [42]; (**c**) Renyi [43]; (**d**) Kapur [4]; (**e**) Masi [44]; (**f**) proposed method (*k* = 1, *t* = 45; *k* = 2, *t* = 94, 95; and *k* = 3, *t* = 123, 124, 125).

Based on Figure 12, we analyzed the segmentation performance of various thresholding methods for *Image4*, comparing the results against the ground truth illustrated in Figure 9. The Shannon and Tsallis entropy models exhibit significant limitations, particularly at *k* = 1, where the segmentation is noisy, and object boundaries are fragmented and unclear. Increasing the number of thresholds (*k* = 2, 3) slightly enhances segmentation, but these models still struggle to distinctly isolate the foreground objects (elephants), leading to excessive over-segmentation in the background regions. The Renyi and Kapur entropy models also show similar limitations. At *k* = 1, these methods produce fragmented regions within areas of interest, resulting in an inconsistent representation of the objects. While the segmentation quality improves at higher thresholds (*k* = 2, 3), these methods still introduce unclear regions, failing to achieve robust segmentation. On the other hand, the Masi entropy model performs better in segmenting the objects compared to the aforementioned methods. It provides more accurate object boundaries at k = 1 and demonstrates enhanced segmentation quality with higher thresholds (*k* = 2, 3). However, Masi occasionally introduces some unclear regions in the foreground. The proposed method consistently achieves superior segmentation performance compared to the existing entropy-based techniques. At a single threshold (*k* = 1), it closely aligns with the ground truth by effectively isolating the elephants and significantly minimizing noise in the background. As the number of thresholds increases (*k* = 2, 3), the proposed method maintains the integrity of the segmented objects while avoiding the over-segmentation issues observed in other methods. Similar to the Masi entropy model, minor fragmented regions are introduced at higher thresholds; however, the proposed approach remains the most reliable and coherent method for accurately segmenting the objects and preserving background consistency.

**Figure 13:**
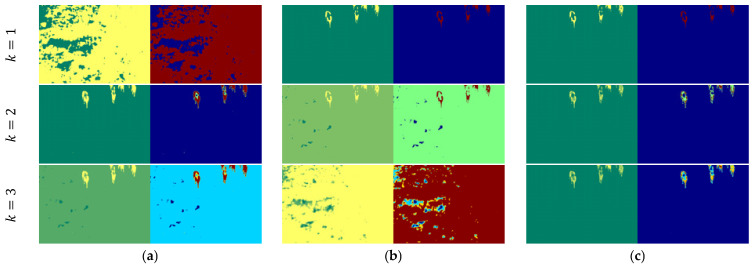

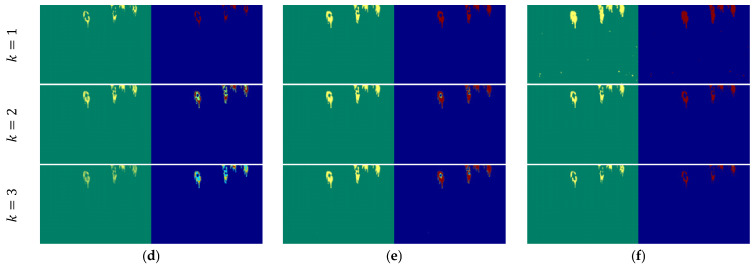
Thresholding results for *Image5*: (**a**) Shannon [48]; (**b**) Tsallis [42]; (**c**) Renyi [43]; (**d**) Kapur [4]; (**e**) Masi [44]; (**f**) proposed method (*k* = 1, *t* = 50; *k* = 2, *t* = 98, 99; and *k* = 3, *t* = 128, 129, 130).

Based on Figure 13, the thresholding results for *Image5* reveal notable differences in performance across the evaluated methods. Shannon entropy fails to segment the objects effectively at *k* = 1. While increasing the number of thresholds (*k* = 2, 3) slightly improves the separation between objects and the background, the segmentation remains suboptimal with fragmented and inconsistent regions. Tsallis entropy demonstrates moderate improvements in object isolation with the initial threshold. However, it fails to deliver robust segmentation, as increasing the threshold level introduces noticeable fragmentation within the objects of interest, compromising their structural integrity. The Renyi, Kapur, and Masi entropy models achieve moderate success in segmenting the objects, with slightly better separation between the foreground and background. Despite this, they suffer from inconsistencies and fail to produce clear object boundaries, resulting in segmented regions that lack precision. In comparison, the proposed method demonstrates superior performance relative to the other approaches. At *k* = 1, it accurately isolates the objects with well-defined boundaries and minimal noise, closely matching the ground truth. As the number of thresholds increases (*k* = 2, 3), the proposed method consistently maintains the integrity of the segmented objects while effectively avoiding over-segmentation. However, similar to the Renyi, Kapur, and Masi entropy models, it introduces slightly unclear segmented regions at higher threshold levels. It is evident that the proposed method demonstrates superior performance with a single threshold, effectively segmenting the regions of interest with high accuracy. This approach not only ensures precise object isolation but also achieves this with reduced computational complexity compared to other methods, making it an efficient and reliable solution for segmentation tasks.

**Figure 14:**
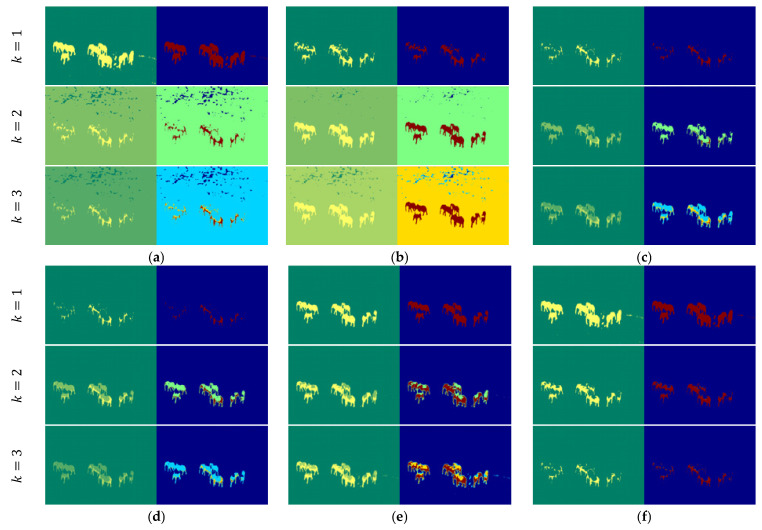
Thresholding results for *Image6*: (**a**) Shannon [48]; (**b**) Tsallis [42]; (**c**) Renyi [43]; (**d**) Kapur [4]; (**e**) Masi [44]; (**f**) proposed method (*k* = 1, *t* = 47; *k* = 2, *t* = 97, 98; and *k* = 3, *t* = 127, 128, 129).

Figure 14 shows the thresholding results for Image6 using various entropy-based methods at different threshold levels (*k* = 1, 2, 3). The Tsallis, Renyi, and Kapur entropy models aim to enhance segmentation by increasing the number of thresholds; however, they do not provide a clear and consistent delineation of the regions of interest (elephants). While the Shannon method performs adequately with a single threshold, it struggles to maintain precision as the number of thresholds increases, resulting in significant noise and fragmented areas. The Masi entropy method demonstrates improved segmentation performance by delivering more accurate regions, though some slight incompleteness remains. As the number of thresholds increases, the accuracy of the segmented results improves, but this comes with greater computational complexity. In contrast, the proposed method consistently outperforms other approaches, especially at *k* = 1, where it achieves superior segmentation accuracy with clearly defined and coherent regions of interest. Although the method introduces slightly ambiguous regions at higher threshold levels, its performance remains competitive. The proposed method with a single threshold excels in efficiently segmenting regions of interest, offering high accuracy while minimizing computational complexity.

**Figure 15:**
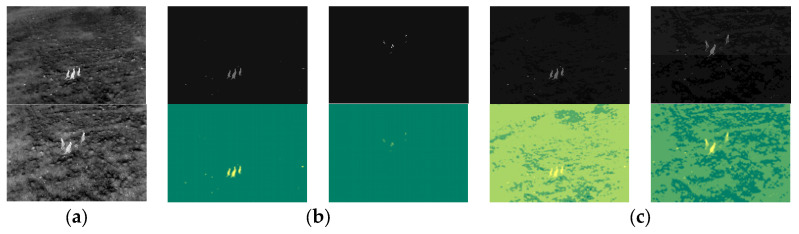
Comparison of a thermal image: (**a**) original, (**b**) single thresholding, and (**c**) multilevel thresholding.

To further clarify the choice between single-threshold and multi-threshold segmentation, we emphasize that the number of optimal thresholds depends on the complexity of the image. Multilevel thresholding is advantageous when an image contains multiple regions of interest with distinct intensity distributions. In complex images, where foreground and background intensities overlap, multilevel thresholding can better distinguish between different objects or regions. This is especially useful for images with heterogeneous thermal distributions, where varying intensity levels correspond to different temperatures or materials. However, single-threshold segmentation is often more effective in high-contrast scenarios. When an image features a clear foreground–background distinction (such as elephants against a relatively uniform thermal background), a single threshold can be sufficient to achieve optimal segmentation. The use of multiple thresholds in these cases may introduce redundant segmentation, increasing computational overhead without significant improvements in accuracy, as illustrated in Figure 15.

## 5. Conclusions

This paper presents A-Entropy, initiated by Agaian, a novel adaptive unsupervised entropy framework designed for the robust segmentation of challenging thermal images. By addressing the limitations of conventional entropy-based methods, A-Entropy incorporates a three-stage process: adaptive preprocessing, A-Entropy-driven thresholding, and postprocessing refinement. This approach effectively enhances the visibility of regions of interest while mitigating noise and artifacts inherent in thermal imagery.

Evaluated on the BIRDSAI dataset, A-Entropy demonstrates significant improvements over established methods such as Shannon, Tsallis, and Kapur. Notably, it achieves 8–12% higher Sørensen–Dice scores and surpasses 0.99 accuracy with a single threshold (*k* = 1), highlighting its efficiency and precision. Visual analysis further confirms its superior ability to preserve structural details and delineate accurate boundaries, even in complex scenes, outperforming competitors like Masi and Renyi.

A-Entropy is adaptable to diverse applications, including medical diagnostics, energy efficiency monitoring, and surveillance. While minor fragmentation at higher thresholds warrants further investigation, this study establishes A-Entropy as a powerful tool for precise and efficient thermal image segmentation, striking a balance between accuracy and computational cost.

## Figures and Tables

**Table 1:** Comparison of meta-heuristic optimization techniques for threshold selection.

Aspect	Genetic Algorithm (GA) [45]	Particle Swarm Optimization (PSO) [46]
Common Goals	Find optimal threshold values. Reduce computational complexity. Avoid local optima. Handle multi-dimensional threshold problems.	Identify optimal threshold values. Minimize computational complexity. Prevent local optima. Address multi-dimensional threshold issues.
Inspiration	Natural selection and genetic evolution.	Social behavior of bird flocking or fish schooling.
Advantages	Excellent at exploring large search spaces. Handles non-linear, non-differentiable problems. Maintains population diversity. Easily parallelizable. Effective for multi-threshold problems.	Simpler implementation. Fewer parameters to tune. Faster convergence for many problems. Efficiently handles continuous optimization. Less sensitive to initialization.
Disadvantages	Requires careful parameter tuning. Convergence can be slow. Computationally intensive. No guarantee of global optimum. Performance depends on fitness function.	Prone to premature convergence. Less effective at full space exploration. Performance degrades with dimensionality. More easily trapped in local optima. Still requires parameter selection.
Key Parameters	Population size, mutation rate, crossover rate, selection method	Inertia weight, cognitive/social acceleration coefficients, swarm size
Application to Thresholding	Well suited for complex multilevel thresholding.	Efficient for continuous threshold optimization.

**Table 2:** Entropy-based metric values for images described in Figure 1.

Image	σ	Shannon [48]	Tsallis [42]	Renyi [43]
Original image	28.7754	6.8363	0.9895	4.5582
Image with pixels shuffled row-wise	28.7754	6.8363	0.9895	4.5582
Image with pixels shuffled column-wise	28.7754	6.8363	0.9895	4.5582
Fully shuffled image	28.7754	6.8363	0.9895	4.5582

**Table 3:** Block-based metric values for images depicted in Figure 1.

Image	EME	EMEE	AME	AMEE	Proposed
Original image	14.5007	0.7858	11.1357	0.4324	0.7076
Image with pixels shuffled row-wise	31.5322	20.5739	13.7218	0.6765	0.8706
Image with pixels shuffled column-wise	31.1548	20.8945	13.7203	0.6763	0.8707
Fully shuffled image	14.5007	0.7858	11.1357	0.4324	0.7076

**Table 4:** Kernel-based metric values for images depicted in Figure 2.

Image	DoE	EME	EMEE	AME	AMEE	Proposed
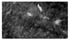 *Image1*	0%	15.5717	0.9833	12.2236	0.5241	0.3245
	25%	16.0210	1.0582	12.6684	0.5666	0.3268
	50%	16.3847	1.1297	12.9094	0.5906	0.3292
	75%	16.7365	1.2205	13.0533	0.6055	0.3312
	100%	17.0714	1.3254	13.1471	0.6156	0.3332
* 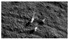 * *Image2*	0%	15.3141	0.9125	12.2881	0.5324	0.3271
	25%	15.6861	0.9682	12.6490	0.5667	0.3288
	50%	16.0095	1.0253	12.8522	0.5867	0.3304
	75%	16.3286	1.0948	12.9693	0.5989	0.3317
	100%	16.6670	1.1881	13.0366	0.6064	0.3327
* 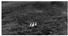 * *Image3*	0%	15.0002	0.8751	11.6894	0.4758	0.3084
	25%	15.4134	0.9405	12.3179	0.5325	0.3114
	50%	15.7595	1.0040	12.6592	0.5648	0.3143
	75%	16.0816	1.0770	12.8633	0.5852	0.3172
	100%	16.3864	1.1613	13.0039	0.5996	0.3200

**Table 5:** Metric descriptions and formulations.

Metric	Description	Mathematical Formulations
Accuracy	Measures the overall proportion of correctly classified pixels, including foreground and background. A general measure of classification performance.	$Accuracy=\frac{TP+TN}{TP+TN+FP+FN}$
Boundary F1 (BF) Score	Evaluates how well predicted boundaries match ground-truth edges, using F1 score principles at the object boundary level. This is critical for applications requiring precise contour alignment.	$BF=\frac{2-Prcision-Recall}{Precision+Recall}$
Sørensen–Dice Similarity Coefficient (DSC)	Measures the overlap between predicted and ground-truth regions, emphasizing correct segmentation of object areas. Also known as the Dice coefficient or F1 score.	$DSC=\frac{2TP}{2TP+FP+FN}$
Jaccard Similarity (IoU)	Assesses the ratio between the intersection and union of the predicted and ground-truth masks. This is useful for understanding overall spatial accuracy.	$IoU=\frac{TP}{TP+FP+FN}$
Precision	Indicates the proportion of correctly predicted positives among all positive predictions, representing prediction reliability.	$Precision=\frac{TP}{TP+FP}$
Recall (Sensitivity)	Measures the proportion of correctly predicted positives among all actual positives, indicating detection completeness.	$Recall=\frac{TP}{TP+FN}$

TP (true positive) represents the number of pixels that have been correctly classified or segmented, FP (false positive) represents the number of background pixels that have been incorrectly classified as foreground (often due to misalignment), FN (false negative) denotes the number of foreground pixels that have been misclassified as background, and TN (true negative) indicates the number of background pixels that have been correctly identified as background.

**Table 6:** Advantages and disadvantages of metrics.

Metric	Advantages	Disadvantages
Accuracy	Simple and intuitive. Effective for balanced datasets.	Misleading for imbalanced data. Does not reflect boundary precision or spatial overlap. May mask poor performance for minority classes.
Boundary F1 (BF) Score	Sensitive to boundary alignment. Critical for contour-based tasks.	Computationally intensive. Less informative for overall region overlap.
Sørensen–Dice Similarity Coefficient (DSC)	Balances false positives and false negatives. Effective for region overlap. Commonly used in medical segmentation.	Less sensitive to boundary errors. Can be inflated in images with large background areas.
Jaccard Similarity (IoU)	Directly measures region overlap. Effective for sparse or imbalanced data. Useful for fair comparison of models.	More sensitive to misclassifications than DSC. Less intuitive interpretation.
Precision	Highlights false positives. Important in false-alarm-sensitive tasks.	Ignores false negatives. May overestimate performance if recall is low.
Recall (Sensitivity)	Highlights missed detections. Essential in completeness-focused tasks.	Ignores false positives. May encourage over-segmentation.

**Table 7:** Accuracy.

Entropy Method	Image	Number of Thresholds		
		*k* = 1	*k* = 2	*k* = 3
Shannon [48]	Image4	**0.9852**	0.8247	0.8751
	Image5	0.1861	**0.9979**	0.0297
	Image6	**0.9964**	0.0952	0.0952
Tsallis [42]	Image4	**0.9802**	0.0482	0.0482
	Image5	**0.9887**	0.0297	0.0297
	Image6	**0.9601**	0.0756	0.0756
Renyi [43]	Image4	0.9734	0.9871	**0.9938**
	Image5	0.9877	0.9937	**0.9971**
	Image6	0.9458	0.9770	**0.9899**
Kapur [4]	Image4	0.9703	0.9663	**0.9880**
	Image5	0.9857	**0.9955**	0.9955
	Image6	0.9401	**0.9841**	0.9841
Masi [44]	Image4	0.9924	0.9951	**0.9956**
	Image5	0.9945	0.9964	**0.9974**
	Image6	0.9841	0.9957	**0.9978**
Proposed	Image4	**0.9997**	0.9847	0.9751
	Image5	**0.9990**	0.9918	0.9877
	Image6	**0.9978**	0.9672	0.9458

**Table 8:** BF (Boundary F1) score.

Entropy Method	Image	Number of Thresholds		
		*k* = 1	*k* = 2	*k* = 3
Shannon [48]	Image4	**0.9233**	0.3676	0.4245
	Image5	0.0819	**0.9862**	0.0673
	Image6	**0.9263**	0.1513	0.1513
Tsallis [42]	Image4	**0.8632**	0.0000	0.0000
	Image5	**0.9227**	0.0673	0.0673
	Image6	**0.8818**	0.2028	0.2028
Renyi [43]	Image4	0.7601	0.9380	**0.9788**
	Image5	0.9061	0.9638	**0.9986**
	Image6	0.8447	0.9510	**0.9892**
Kapur [4]	Image4	0.7109	0.6389	**0.9423**
	Image5	0.8861	**0.9877**	0.9877
	Image6	0.8093	**0.9801**	0.9801
Masi [44]	Image4	0.9698	0.9893	**0.9944**
	Image5	0.9733	**0.9965**	0.9964
	Image6	0.9801	**0.9917**	0.9541
Proposed	Image4	**0.9821**	0.9179	0.7939
	Image5	0.8964	**0.9444**	0.9061
	Image6	**0.9412**	0.9151	0.8447

**Table 9:** Sørensen–Dice Similarity.

Entropy Method	Image	Number of Thresholds		
		*k* = 1	*k* = 2	*k* = 3
Shannon [48]	Image4	**0.8148**	0.3508	0.4314
	Image5	0.0550	**0.9532**	0.0465
	Image6	**0.9755**	0.1367	0.1367
Tsallis [42]	Image4	**0.7351**	0.0905	0.0905
	Image5	**0.6873**	0.0465	0.0465
	Image6	**0.6136**	0.1342	0.1342
Renyi [43]	Image4	0.6093	0.8425	**0.9298**
	Image5	0.6497	0.8451	**0.9353**
	Image6	0.3907	0.8088	**0.9239**
Kapur [4]	Image4	0.5442	0.4478	**0.8551**
	Image5	0.5655	**0.8950**	0.8950
	Image6	0.2814	**0.8749**	0.8749
Masi [44]	Image4	0.9125	0.9450	**0.9517**
	Image5	0.8673	0.9172	**0.9408**
	Image6	0.8749	0.9690	**0.9846**
Proposed	Image4	**0.9966**	0.8067	0.6440
	Image5	**0.9799**	0.7890	0.6497
	Image6	**0.9850**	0.7035	0.3907

**Table 10:** Jaccard Similarity.

Entropy Method	Image	Number of Thresholds		
		*k* = 1	*k* = 2	*k* = 3
Shannon [48]	Image4	**0.6874**	0.2127	0.2750
	Image5	0.0283	**0.9106**	0.0238
	Image6	**0.9523**	0.0733	0.0733
Tsallis [42]	Image4	**0.5218**	0.0474	0.0474
	Image5	**0.5235**	0.0238	0.0238
	Image6	**0.4426**	0.0719	0.0719
Renyi [43]	Image4	0.4381	0.7278	**0.8689**
	Image5	0.4811	0.7318	**0.8785**
	Image6	0.2428	0.6789	**0.8586**
Kapur [4]	Image4	0.3739	0.2885	**0.7469**
	Image5	0.3943	**0.8099**	0.8099
	Image6	0.1637	**0.7776**	0.7776
Masi [44]	Image4	0.8390	0.8957	**0.9079**
	Image5	0.7656	0.8470	**0.8882**
	Image6	0.7776	0.9398	**0.9696**
Proposed	Image4	**0.9933**	0.6761	0.4749
	Image5	**0.9607**	0.6515	0.4811
	Image6	**0.9704**	0.5426	0.2428

**Table 11:** Precision.

Entropy Method	Image	Number of Thresholds		
		*k* = 1	*k* = 2	*k* = 3
Shannon [48]	Image4	**1.0000**	0.2127	0.2750
	Image5	0.0283	**0.9969**	0.0238
	Image6	**0.9523**	0.0733	0.0733
Tsallis [42]	Image4	**1.0000**	0.0474	0.0474
	Image5	**1.0000**	0.0238	0.0238
	Image6	**1.0000**	0.0719	0.0719
Renyi [43]	Image4	**1.0000**	1.0000	1.0000
	Image5	**1.0000**	1.0000	0.9998
	Image6	**1.0000**	1.0000	1.0000
Kapur [4]	Image4	**1.0000**	1.0000	1.0000
	Image5	**1.0000**	1.0000	1.0000
	Image6	**1.0000**	1.0000	1.0000
Masi [44]	Image4	**1.0000**	1.0000	1.0000
	Image5	**1.0000**	1.0000	0.9992
	Image6	**1.0000**	0.9985	0.9780
Proposed	Image4	0.9933	**1.0000**	1.0000
	Image5	0.9607	**1.0000**	1.0000
	Image6	0.9704	**1.0000**	1.0000

**Table 12:** Recall.

Entropy Method	Image	Number of Thresholds		
		*k* = 1	*k* = 2	*k* = 3
Shannon [48]	Image4	0.6874	**1.0000**	1.0000
	Image5	**1.0000**	0.9132	1.0000
	Image6	**1.0000**	1.0000	1.0000
Tsallis [42]	Image4	0.5812	**1.0000**	1.0000
	Image5	0.5235	**1.0000**	1.0000
	Image6	0.4426	**1.0000**	1.0000
Renyi [43]	Image4	0.4381	0.7278	**0.8689**
	Image5	0.4811	0.7318	**0.8787**
	Image6	0.2428	0.6789	**0.8586**
Kapur [4]	Image4	0.3739	0.2885	**0.7469**
	Image5	0.3943	**0.8099**	0.8099
	Image6	0.1637	**0.7776**	0.7776
Masi [44]	Image4	0.8390	0.8957	**0.9079**
	Image5	0.7656	0.8470	**0.8889**
	Image6	0.7776	0.9412	**0.9912**
Proposed	Image4	**1.0000**	0.6761	0.4749
	Image5	**1.0000**	0.6515	0.4811
	Image6	**1.0000**	0.5426	0.2428

## Data Availability

The data presented in this study are available on request from the corresponding authors.
